# Zebrafish as a model to understand autophagy and its role in neurological disease^☆^

**DOI:** 10.1016/j.bbadis.2011.01.004

**Published:** 2011-04

**Authors:** Angeleen Fleming, David C. Rubinsztein

**Affiliations:** a Department of Medical Genetics, University of Cambridge, Cambridge Institute for Medical Research, Addenbrooke's Hospital, Hills Road, Cambridge CB2 0XY, UK; b Department of Physiology, Development and Neuroscience, University of Cambridge, Downing Street, Cambridge CB2 3EG, UK

**Keywords:** Zebrafish, Autophagy, Neurodegeneration

## Abstract

In the past decade, the zebrafish (*Danio rerio*) has become a popular model system for the study of vertebrate development, since the embryos and larvae of this species are small, transparent and undergo rapid development *ex utero*, allowing *in vivo* analysis of embryogenesis and organogenesis. These characteristics can also be exploited by researchers interested in signaling pathways and disease processes and, accordingly, there is a growing literature on the use of zebrafish to model human disease. This model holds great potential for exploring how autophagy, an evolutionarily conserved mechanism for protein degradation, influences the pathogeneses of a range of different human diseases and for the evaluation of this pathway as a potential therapeutic strategy. Here we summarize what is known about the regulation of autophagy in eukaryotic cells and its role in neurodegenerative disease and highlight how research using zebrafish has helped further our understanding of these processes.

## Research highlights

► Zebrafish has become a popular model system for the study of vertebrate development. ► There is a growing literature on the use of zebrafish to model human disease. ► This model has potential for exploring how autophagy affects disease pathogenesis. ► This review considers how zebrafish may help further understanding of these processes.

## 1 Introduction

### 1.1 Protein degradation pathways

**Fig. 1:**
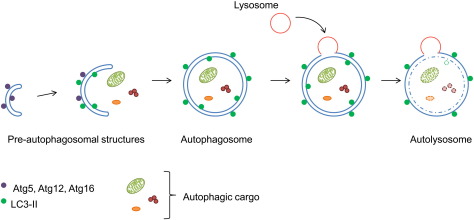
Schematic model of autophagy. Pre-autophagosomal structures form within the cytoplasm. Atg5, Atg12 and Atg16l proteins are recruited to the structure and facilitate elongation. The elongated membranes enwrap a region of the cytoplasm and its contents in a double-membraned autophagosome. Lysosomes ultimately fuse with autophagosomes releasing lysosomal hydrolases into the vesicle resulting in the degradation of its contents.

Efficient degradation of proteins is essential to maintain normal cell homeostasis. In eukaryotes, there are two main degradative pathways; the ubiquitin-proteasome pathway and the autophagy-lysosome pathway. The proteasome is a barrel-shaped multi-subunit protein complex, the core of which contains the components necessary for proteolysis. Proteins are generally targeted to the proteasome after they are tagged by a chain of four or more covalently bonded ubiquitin molecules. In addition to the specificity imposed by the requirement of an ubiquitination signal, the narrow core of the proteasome barrel limits the size of proteins that can be degraded via this pathway. Typically, short-lived and long-lived cytosolic and nuclear proteins are degraded by the proteasome. In contrast, macroautophagy (from hereon referred to as autophagy) can mediate non-specific, bulk degradation of long-lived cytosolic proteins and organelles. Autophagic degradation requires the formation of a double-membraned vesicle, the autophagosome, around a portion of the cytoplasm. Ultimately, autophagosomes fuse with lysosomes to form autolysosomes, acidic compartments in which lyosomal hydrolases degrade any proteins contained within the vesicle (see Fig. 1). Autophagy occurs at a basal level in mammalian cells, but is upregulated in response to various physiological stress conditions e.g. starvation. While it is primarily a mechanism to ensure cell survival, there is increasing evidence for the importance of autophagy as a mechanism for cell death, particularly in insect metamorphosis [1].

### 1.2 Zebrafish models of neurodegeneration

The optical clarity, speed of development, and fecundity of zebrafish have made them a popular vertebrate model for the study of developmental biology, and, more recently, as an animal model to study disease processes [2,3]. The creation of transgenic zebrafish is relatively straightforward [4–7], and has been used to successfully generate models of a range of human neurodegenerative disorders. Diseases caused by dominant mutations can be modeled by expressing the mutated human gene under the control of a zebrafish promoter. Such an approach has been used to model polyglutamine expansion diseases, like Huntington's disease [8], tauopathy [9–11] and amyotrophic lateral sclerosis (ALS) [12]. Furthermore, since zebrafish larvae are transparent, fluorescent transgene constructs can be used in a variety of ways to examine disease pathogenesis, *in vivo*. For example, reporter lines where particular neurons are fluorescently labeled have been used to investigate the sensitivity of monoaminergic neurons to the neurotoxin MPTP [13]. Bi-directional transgenic constructs have been used to create lines in which a fluorescent protein signal is expressed with the same spatial and temporal control as the disease-causing protein [11]. Similarly, direct fusion of a fluorescent protein to the disease-causing transgene has been employed as a read-out of transgene expression, but can also be used as a marker for protein aggregation. Such an approach has been used to examine huntingtin aggregate clearance *in vivo*, as a method for validating novel therapeutic strategies [8]. In addition to the creation of stable transgenic lines to model dominant genetic mutations, transient over-expression techniques have been used to this end. In such studies, injection of DNA or mRNA into fertilized eggs results in transient expression of the disease-causing protein during embryogenesis and in early larval stages [14–18]. Although there is an inherent level of variability in gene expression, this method has proved powerful for the study of disease modifiers in models of polyglutamine disease [14] and motor neuron disease [16–19] and in the evaluation of therapeutic strategies [15].

Loss-of-function models of neurodegeneration have also been widely explored in zebrafish (see [20] for review). The most widely used technique for the study of loss-of-function is that of transient knockdown using antisense technologies. Morpholino oligonucleotides are the most commonly used, validated and accepted antisense technique in zebrafish [21], although other antisense technologies, such as peptide nucleic acid mimics (gripNAs) [22], are now gaining popularity. Recently, spatial and temporal control of morpholino knockdown has been described, by combining a neutralizing strand with the morpholino oligonucleotide that is photocleavable by irradiation with UV light [23]. Transient knockdown techniques have been used to develop zebrafish models of Parkinson's disease [24–29], ALS [30] and spinal muscular atrophy (SMA) [31–33]. In addition to their use for developing disease models for loss-of function disorders, antisense knockdown technologies have been used to investigate normal gene function and elucidate novel signaling pathways in a range of neurodegenerative disorders including Huntington's disease [34–37], Alzheimer's disease [38–41] and SMA [32,42–44] and to investigate the role of progranulin and TDP-43 in the pathogenesis of ALS and frontotemporal lobe dementia [17–19,45].

The limitation of such antisense techniques is that knockdown is transient, usually only lasting to 5–7 d.p.f. Therefore efforts have focused on the development of methods for targeted gene knockdown in zebrafish [46–48], with the recent reports of zinc finger nuclease (ZFN) technology holding promise for the widescale and specific generation of heritable loss-of-function alleles [49–51]. In addition, random mutagenesis screens [52,53] have yielded numerous mutant zebrafish lines in which the mutated gene is implicated in a human disease, e.g. muscular dystrophy [54–57], and it is hoped that the continuing screening of mutagenized libraries (e.g. TILLING—Targeting Induced Local Lesions in Genomes) will yield mutations in specific genes of relevance to neurodegenerative disorders.

In addition to the ease of genetic manipulation, zebrafish are also highly amenable to pharmacological manipulation [58]. This allows up and down-regulation of cell signaling pathways by chemical agonists and antagonists, in addition to the use of zebrafish disease models to test therapeutic strategies [8,11] and perform compound screens [15]. The zebrafish offers advantages over equivalent rodent models, since the manifestation of disease phenotypes is typically more rapid than in their rodent equivalent, larvae can be arrayed in multi-well plates, and compound requirement is small. Since zebrafish are highly amenable to both genetic modification [59,60] and direct compound screening in a tractable fashion [61], they are potentially a powerful tool for the investigation of the autophagy pathway and its role in neurodegeneration. The remainder of this article is dedicated to the application of such zebrafish models to the study of autophagy.

### 1.3 The molecular control of autophagy in eukaryotes

**Fig. 2:**
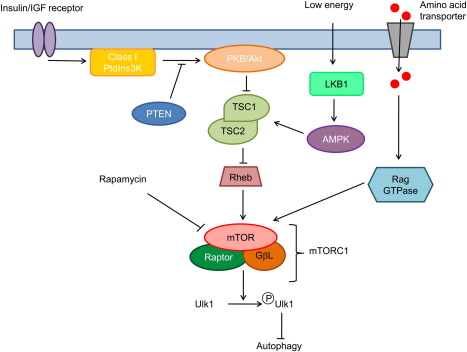
Simplified schematic of the regulatory pathways controlling autophagy. Additional regulatory pathways exist in mammalian cells (reviewed in [62,73]) but have yet to be investigated in zebrafish.

Under normal conditions, autophagy occurs at basal levels, but can be induced rapidly in response to stress conditions and extracellular signals. Target of rapamycin (TOR), a serine/threonine protein kinase, is a central component controlling autophagy, integrating signals from multiple upstream pathways and inhibiting autophagy (see Fig. 2). The regulatory pathways controlling autophagy are well described elsewhere [62], hence this review focuses on aspects of the pathway where the zebrafish model has or could be employed to further our understanding of this process.

mTOR forms two distinct complexes (mTORC1 and mTORC2), which vary both in their subunit components and their function. The mTORC1 complex consists of 3 subunits: mTOR, G protein β-subunit-like (mSLT8) and the regulator-associated protein mTOR (Raptor). Under normal conditions, the mTORC1 complex blocks autophagy by phosphorylating Ulk1 [63–65], but this inhibitory activity is repressed by rapamycin treatment (a specific TOR inhibitor) or starvation conditions, leading to an upregulation of autophagy. mTORC1 is itself inhibited by the action of the tuberous sclerosis complex 1 and 2 proteins (TSC1 and TSC2), which together form a complex (TSC1/2) (Fig. 2). The mTORC2 complex consists of 4 subunits: mTOR, mSLT8, Rictor (rapamycin-insensitive companion of mTOR) and mSin1 (mitogen-activated-protein-kinase-associated protein 1). The mTORC2 complex regulates actin cytoskeleton dynamics and is not involved directly in the regulation of autophagy [66].

The conservation of TOR signaling pathway has been explored in zebrafish using both pharmacological manipulation and morpholino gene knockdown [67]. Zebrafish have a single homologue of mTOR which, although expressed ubiquitously during early embryogenesis, becomes localized to the head and developing gut between 35 and 57 hours post-fertilization (h.p.f.). Treatment of zebrafish with rapamycin during early embryogenesis resulted in developmental delay, but did not cause any overt defects. However, longer treatment resulted in specific defects in the growth and morphogenesis of the zebrafish gut. Morpholino knockdown of zebrafish mTOR, raptor and S6 kinase (an mTOR effector that regulates translation but not autophagy) resulted in defects in the development of the digestive tract that phenocopy those observed with rapamycin treatment, whereas knockdown of rictor had only minimal effects in gut growth and morphogenesis [67]. This study demonstrates a critical role for TOR via the TORC1 complex in vertebrate intestinal development. Importantly, since TOR regulates many processes besides autophagy, many of these consequences of mTOR inhibition may be autophagy-independent. The equivalent studies have not been performed in mouse embryos, since knockdown of mTOR results in embryonic lethality [68]. This highlights the advantages that the zebrafish offers over the mouse in the study of gene knockdown effects, as embryonic events can be more readily visualized. However, a recent study using morpholino knockdown of the autophagy-related gene gabarap reported microcephaly and jaw defects in zebrafish morphants [69], whereas gabarap knockout mice are phenotypically normal [70], suggesting that further studies are needed to determine whether the roles of autophagy in mammalian development are conserved in non-mammalian vertebrates. Many more components of the autophagy pathway and aspects of TOR-independent autophagy regulation remain unexplored in zebrafish. A summary of the zebrafish homologs of selected mammalian components of the autophagy regulatory pathway are listed in Table 1. The ability to perform gene knockdown and to temporally control signaling pathways using pharmacological inhibitors, as described above, highlight the potential of this model for the investigation of autophagy regulation.

### 1.4 Tools for assessing autophagy in zebrafish

In addition to understanding the molecular control of autophagy and its conservation between zebrafish and mammals, it is important to examine the onset of expression of the pathway components. The formation of autophagosomes is assessed by the conversion of LC3-I to LC3-II, since LC3-II is specifically associated with autophagosomes. In contrast to the mouse embryo, where LC3-II is observed in oocytes [71], He et al. demonstrated that zebrafish LC3-II was only detectable from 32 h.p.f. onwards. Although RT-PCR analysis demonstrated the presence of transcripts of the autophagy genes beclin and lc3 at 0 h.p.f., ulk1a and ulk1b (identified as putative zebrafish homologs of the single mammalian ulk1 autophagy gene) and atg9a and atg9b were not expressed until at least 23 h.p.f. [72]. The later expression of these autophagy genes may explain why autophagosome formation (as measured by LC3-II) is delayed in the zebrafish embryo relative to mammalian embryos, and also raises questions about the importance of autophagy in early embryogenesis in this organism.

Gene knockdown studies could be of great value in dissecting the roles of individual pathway components (listed in Table 1) in the regulation of autophagy. This approach has been widely adopted *in vitro*, using siRNA and shRNA (comprehensively reviewed in [73]). However, little work has been performed to confirm these findings *in vivo*. Here, gene knockdown in zebrafish may offer advantages over knockout mouse studies, which are, by comparison, lengthy and costly. Such an approach was recently employed by Dowling et al. [74] investigating the work of myotubularins in the regulation of autophagy and in the pathogenesis of centronuclear myopathy. Myotubularins (MTM) and myotubularin-related proteins (MTMR) are family of phosphatases that dephosphorylate phosphinositides. Using siRNA, MTMR14 (also called Jumpy) was previously demonstrated to act as a suppressor of autophagy *in vitro*, since knockdown resulted in an increase in autophagosome formation [75]. In zebrafish, knockdown of MTMR14 was shown to cause a similar increase in autophagy (as measured by LC3-II levels) and double knockdown studies with MTM1 resulted in a phenotype reminiscent of human centronuclear myopathy [74].

Another valuable tool in the study of autophagy is the measurement of LC3-II levels. Western blotting to detect LC3-II can be used to determine the number of autophagosomes and to measure changes in autophagic flux. Using such an approach, He et al. [72] demonstrated that rapamycin increases autophagosome synthesis in larval zebrafish, compatible with its effects in other organisms. In addition, the number of autophagosomes (LC3-II levels) in zebrafish can be enhanced by treatment with lysosomal inhibitors such as pepstatinA, E64d [72], or ammonium chloride [76]. These agents reduce the acidity of the lysosome and thereby decrease autophagosome/LC3 degradation. Measuring LC3-II levels in the presence or absence of lysosomal inhibitors provides a useful tool for measuring autophagic flux in cells [77] and has recently been applied to *in vivo* investigations in zebrafish to assess the effects of antioxidants on autophagy [76]. To further study the process of autophagy in zebrafish, He et al. [72] generated transgenic reporter lines expressing GFP-tagged lc3 and Gabarap, and demonstrated that distribution of the fluorescently tagged proteins changed appropriately following treatment with a variety of known autophagy inducing and inhibiting agents. These lines will be of value in future studies for the validating the mechanism of action of compounds and could be used in combination with the disease models described elsewhere in this issue to evaluate the role of autophagy in the pathogenesis of neurodegeneration.

### 1.5 Autophagy as a therapeutic strategy for neurodegenerative diseases

A common feature of many late-onset neurodegenerative disorders, including Parkinson's disease, Alzheimer's disease, Huntington's disease, tauopathies, and various spinocerebellar ataxias is the accumulation of misfolded or aggregating proteins within the cell. Under normal conditions, the basal rate of autophagy is not sufficient to prevent the accumulation of cytoplasmic aggregate-prone proteins aggregates over many years. However, induction of autophagy by treatment with rapamycin has proven effective in enhancing the clearance of aggregate-prone proteins *in vitro* [78–80] and *in vivo*, in *Drosophila* models of Huntington's disease and tauopathy [79,80] and mouse models of Huntington's disease and spinocerebellar ataxia type 3 [79,81]. These studies provide proof-of-principle that upregulation of autophagy may be an effective therapeutic strategy for the clearance of aggregate-prone proteins. While rapamycin has been demonstrated to be effective and is prescribed for chronic use in people, it has side effects that make it desirable to find safer and possibly more specific autophagy inducers that can be used to treat patients for many decades. In some cases, patients may be asymptomatic gene carriers of mutations causing conditions like Huntington's disease, where the objective of the treatment would be to delay onset of disease. Several groups have now identified novel compounds that induce autophagy *in vitro* [82–85], although the challenge remains as to how best to validate these findings *in vivo*. Traditionally, *in vivo* validation has been performed on rodent models. However, large scale screens can produce tens or possibly hundreds of “hits” that require further validation, including *in vivo* testing, in order to select the best therapeutic candidate for further development. Validating all “hit” compounds in rodent models is often not feasible due to the length and cost of trials, in addition to large amounts of compound required for long-term treatment regimes. Here zebrafish models offer a distinct advantage, since many of the neurodegenerative disease models described to date develop disease phenotypes at larval stages [8,9,11]. Williams et al. used such a screening cascade to identify novel inducers of autophagy from a library of FDA-approved drugs. Primary screens were performed using increased clearance of mutant α-synuclein (that causes familial Parkinson's disease) and mutant huntingtin (that causes Huntington's disease) as indicators of increased autophagy. Compounds demonstrated to be effective in cell-based assays were then tested in *Drosophila* and zebrafish models of Huntington's disease [8]. As screening assays become more sophisticated, with a shift towards high-content read-outs, zebrafish models offer great potential for the development of novel screening assays, using, for example, fluorescent reporters or high-throughput behavioral analysis to identify agents that ameliorate the disease phenotype.

### 1.6 Future directions

While this review highlights the potential for zebrafish as a model for the study of autophagy, a number of uncertainties or technical limitations remain and should be considered as priorities for future investigation:

• Gene duplications—the conservation of function between mammalian and zebrafish proteins is unclear for genes where zebrafish possess several homologs (e.g. PTEN) and further work is needed to assess the overlapping and/or non-redundant roles of these. In addition, caution is needed in the interpretation of potentially duplicated sequences identified in genomic databases and it is expected that the ongoing annotation of the zebrafish genome will clarify whether previously reported duplications (e.g. tsc1a and tsc1b [88], ulk1a and ulk1b [72]) are genuine or whether these have arisen from incomplete annotation.
• Targeted gene knockouts—although morpholinos provide a powerful tool for transient gene knockdown, an effective technology for permanent gene knockdown would be desirable for some studies.
• Compound uptake and distribution—while zebrafish offer huge potential for *in vivo* validation of novel therapies, little is known about compound absorption, distribution or metabolism. Of particular relevance to the study of neurological disorders, it is important to consider the timing of zebrafish blood–brain barrier formation and the similarities or differences between this barrier in zebrafish and mammals.

## Figures and Tables

**Table 1:** Zebrafish homologs of key components of the autophagy pathway.

Mammalian gene	Zebrafish homolog(s)	Accession number(s)	RefSeq status	Notes
Akt/Protein kinase B (Akt1)	No sequence homologs for Akt1 identified in Genbank			
AMBRA1	No sequence homologs identified in Genbank			
ATG10	atg10	NM_001037124	Provisional	
ATG12	atg12	XM_694510	Model	Predicted homolog.
ATG16L1	atg16l1	NM_001017854	Provisional	
ATG3	apg3l, autophagy 3-like	NM_200022	Provisional	
ATG4A	atg4a	NM_001024434	Provisional	
ATG4B	atg4b	NM_001089352	Provisional	
ATG4C	atg4c	NM_001002103	Provisional	
ATG4D	LOC795933 autophagy-related 4D-like	XM_001333057	Model	Predicted homolog.
ATG5	atg5	Isoform 1: NM_001009914	Provisional	
		Isoform 2: NM_205618	Provisional	
ATG7	atg7	XM_002663680	Model	Predicted homolog; partial mRNA sequence.
ATG9A	atg9a	NM_001083031	Provisional	
ATG9B	atg9b	NM_001080705	Provisional	
Bcl2	bcl2	NM_001030253	Provisional	
Beclin1	beclin1	NM_200872	Provisional	
Gabarap	gabarap	NM_001013260	Validated	
GβL/MLST8	mlst8	NM_199877	Provisional	
MAP1LC3A	map1lc3a	NM_214739	Provisional	
MAP1LC3B	map1lc3b map1lc3b-like	NM_199604XM_002664472	Provisional Model	Possible gene duplication in zebrafish.
MAP1LC3C	zgc:56565	NM_200298	Provisional	
PTEN	ptena	NM_200708	Provisional	ptena and ptenb encode functional enzymes with spatially distinct expression patterns [86,87].
	ptenb	NM_001001822	Provisional	
Raptor	Rptor (raptor-like)	XM_002662358	Model	Predicted homolog.
Rheb	Rheb	NM_001076748	Validated	
Rictor	Rictor	XM_001921872	Model	Predicted homolog. Homolog previously reported in [67] (XM_685234) has now been removed from Genbank.
SQSTM1	sqstm1	XM_002662358	Provisional	
TOR	mTOR	NM_001077211	Provisional	
TSC1	tsc1a	NM_200052	Provisional	TSC1b reported in [88] now annotated as non-coding RNA.
	(tsc1b)	(NR_023332)	Provisional	
TSC2	tsc2	XM_690820	Model	Predicted homolog.
Ulk1	Ulk1b	XM_002665925	Model	Chromosome 21 (predicted homolog). Two zebrafish homologs (Ulk1a and Ulk1b) reported in [72], based on Blast searches but no accession numbers published.
Ulk2	No sequence homologs identified in Genbank			
UVRAG	uvrag	NM_201069	Provisional	
VPS45	vps45	XM_002665582	Model	
WIPI1	wipi1	NM_200391	Provisional	

Zebrafish homologs of mammalian genes were identified using NCBI Entrez Nucleotide and NCBI Entrez Gene search engines [89,90]. Due to the incomplete nature of these databases, zebrafish homologs for some mammalian genes do not have entries (e.g. AMBRA, Ulk2). However, search tools such as BLAST can be used to identify zebrafish homolog(s). RefSeq status is a useful indicator of the confidence that the homolog has been correctly assigned [91]. Searches correct as of 30th October 2010.
